# Prevalence and risk factors associated with stroke in middle-aged and older Chinese: A community-based cross-sectional study

**DOI:** 10.1038/s41598-017-09849-z

**Published:** 2017-08-25

**Authors:** Yong Gan, Jiang Wu, Shengchao Zhang, Liqing Li, Xiaoxv Yin, Yanhong Gong, Chulani Herath, Naomie Mkandawire, Yanfeng Zhou, Xingyue Song, Xiaozhou Zeng, Wenzhen Li, Qiaoyan Liu, Chang Shu, Zhihong Wang, Zuxun Lu

**Affiliations:** 1 Department of Social Medicine and Health Management, School of Public Health, Tongji Medical College, Huazhong University of Science and Technology, Wuhan, Hubei China; 2 Bao’an Central Hospital of Shenzhen, Shenzhen, Guangdong China; 3 Department of Management, School of Economics and Management, Jiangxi Science and Technology Normal University, Nanchang, Jiangxi China; 4 Department of Epidemiology and Biostatistics, School of Public Health, Tongji Medical College, Huazhong University of Science and Technology, Wuhan, Hubei China; 5 Department of Neurosurgery, Shenzhen Second People’s Hospital, Shenzhen University, Shenzhen, Guangdong China

## Abstract

Although the prevalence of stroke and its risk factors has been widely reported in some Western countries, information on essential stroke parameters is lacking in China, the most populous nation. A community-based cross-sectional study with 8,018 Chinese adults aged ≥40 years was used to determine the prevalence of stroke and associated risk factors. Within the screened population, the prevalence of stroke was 2.21% for both sexes, 1.60% for females, and 3.18% for males. Prevalence increased with age in both sexes (*P* < 0.0001). In a multivariable model, factors significantly associated with stroke were increasing age (odds ratio [OR] = 1.87, 95% CI: 1.58–2.24), male gender (OR = 2.03, 95% CI: 1.42–2.90), family history of stroke (OR = 4.33, 95% CI: 2.89–6.49), history of hyperlipidemia (OR = 1.87, 95% CI 1.31–2.68), history of hypertension (OR = 1.47, 95% CI 1.02–2.12), and physical inactivity (OR = 1.74, 95% CI: 1.16–2.59). The findings indicate that stroke prevalence in middle-aged and older Chinese adults is higher in males than in females, and increases with age in both sexes. Population-based public health intervention programs and policies targeting hyperlipidemia and hypertension control and encouragement of physical activity should be highly prioritized for middle-aged and older adults in Shenzhen, China.

## Introduction

Stroke is a major public health problem, affecting millions of people in both developed and developing countries^1^. Stroke is the first leading cause of death and the most prominent factor related to disability-adjusted life-years in China, with high social and economic costs^2^. In addition, stroke has a high recurrence rate among nonfatal cases due to extremely low treatment rates and poor adherence to essential treatments for secondary prevention^3,4^. Therefore, prevention strategies aimed at reducing stroke incidence are important.

The China National Stroke Prevention Project (CSPP) was launched by the Chinese government as the key national action on stroke prevention and control since 2011. The CSPP aimed to decrease the incidence of stroke in the general population. Some provincial or large city hospitals were designated by the CSPP in the nation, as those hospitals were involved in stroke prevention and control work. Shenzhen Second People’s Hospital, designated a large city hospital by the CSPP, was involved in field work in Shenzhen.

Studies of the prevalence of stroke and its risk factors have been conducted in the general population of people with several different nationalities; however, China is the most populous nation in the world, and no studies have focused on stroke prevalence among the general Chinese population. With stroke becoming a greater public health problem, information on stroke prevalence and predictors in the Chinese population is urgently needed to facilitate future healthcare planning. Using data obtained in Shenzhen from the CSPP, we described the epidemiology and risk factors of stroke based on a community-based survey. To our knowledge, this study is the first to report on the prevalence and risk factors for stroke based on a community-based sample of middle-aged and older adults in China. In light of China national stroke prevention and control goals, the results of this study should inform policy makers about priority areas for strengthening the system for preventing stroke in Shenzhen, China and provide valuable evidence on the topic of international stroke prevention and control.

## Results

The main characteristics of participants with and without stroke are reported in Table 1. A total of 8,018 participants (4,937 females, 61.57%) were investigated in this study. The ages of the participants ranged from 40 to 107 years (mean age of 55.49 and standard deviation (SD) = 9.93).

**Table 1:** Descriptive statistics for sample characteristics according to stroke status.

Characteristics	*N (%)*	Stroke cases *(%)*	Non-cases *(%)*	*P value*
Total	8018 (100.00)	177 (2.21)	7841 (97.79)	
Age, y*	55.49 (9.93)	63.81 (9.55)	55.30 (9.85)	<0.0001
40~	2749 (34.29)	18 (0.65)	2731 (99.35)	<0.0001
50~	2488 (31.03)	33 (1.33)	2455 (98.67)	
60~	2016 (25.14)	78 (3.87)	1938 (96.13)	
70~	765 (9.54)	48 (6.27)	717 (93.73)	
BMI (kg/m^2^)*	24.33 (3.20)	25.08 (3.37)	24.31 (3.19)	0.0015
<25	4968 (61.96)	91 (1.83)	4877 (98.17)	
25–29	2677 (33.39)	75 (2.80)	2602 (97.20)	0.0051
30+	373 (4.65)	11 (2.95)	362 (97.05)	
Gender				
Male	3081 (38.43)	98 (3.18)	2983 (96.82)	<0.0001
Female	4937 (61.57)	79 (1.60)	4858 (98.40)	
Education level				
Primary school or below	2623 (32.71)	65 (2.43)	2558 (97.52)	0.7079
Junior middle school	2096 (26.14)	44 (2.10)	2052 (97.90)	
Senior middle school	1745 (21.76)	37 (2.12)	1708 (97.88)	
College degree or above	1554 (19.38)	31 (1.99)	1523 (98.01)	
Marital status				
Unmarried/widow/divorced	361 (4.50)	12 (3.32)	349 (96.68)	0.1396
Married	7657 (95.50)	165 (2.15)	7492 (97.85)	
Health insurance				
Yes	6196 (82.00)	129 (2.08)	6067 (97.92)	0.1457
No	1360 (18.00)	37 (2.72)	1323 (97.28)	
Family history of stroke				
Yes	704 (9.04)	52 (7.39)	652 (92.61)	<0.0001
No	7084 (90.96)	125 (1.76)	6959 (98.24)	
History of diabetes				
Yes	746 (9.31)	34 (4.56)	712 (95.44)	<0.0001
No	7271 (90.69)	143 (1.97)	7128 (98.03)	
History of hypertension				
Yes	2766 (35.03)	101 (3.65)	2665 (96.35)	<0.0001
No	5130 (64.97)	73 (1.42)	5057 (98.58)	
History of hyperlipidemia				
Yes	2981 (37.18)	104 (3.49)	2977 (96.51)	<0.0001
No	5037 (62.82)	73 (1.45)	4964 (98.55)	
History of heart disease				
Yes	410 (5.11)	29 (7.07)	381 (92.93)	<0.0001
No	7608 (94.89)	148 (1.95)	7460 (98.05)	
History of AF				
Yes	156 (2.01)	12 (7.69)	144 (92.31)	<0.0001
No	7592 (97.99)	133 (1.75)	7459 (98.25)	
Smoking status				
Never	7088 (88.40)	142 (2.00)	6946 (98.00)	<0.0001
Former	141 (1.76)	11 (7.80)	130 (92.20)	
Current	789 (9.84)	24 (3.04)	765 (96.96)	
Alcohol drinking				
Never	6996 (87.25)	153 (2.19)	6843 (97.81)	0.7646
Occasionally	722 (9.00)	17 (2.35)	705 (97.65)	
Frequently	300 (3.74)	7 (2.33)	293 (97.67)	
Physical activity				
Yes	6456 (84.03)	102 (1.58)	6354 (98.42)	<0.0001
No	1227 (15.97)	51 (4.16)	1176 (95.84)	

*Mean ± standard deviation.

Abbreviations: AF, atrial fibrillation; BMI, body mass index.

Of the participants, 177 prevalent cases of stroke were identified (136 ischemic, 27 hemorrhagic, and 14 of undetermined cause). The prevalence rate of stroke among the study population aged ≥ 40 years was 2.21%. Males had a higher prevalence (3.18%) of stroke than females (1.60%). Participants who had a higher prevalence of stroke were older, had slightly higher body mass index (BMI), and were more likely to have a family history of stroke. These participants also had higher rates of smoking and reported less physical activity. Moreover, they were more likely to have a history of hypertension, diabetes, hyperlipidemia and atrial fibrillation (AF). The participants exhibited no significant differences in the prevalence of stroke in terms of marital status, educational level, health insurance status, and alcohol drinking (Table 1).

Additional information regarding the effects of gender on the age-specific prevalence of stroke is shown in Table 2. Prevalence increased with age in both sexes (*P* for trend < 0.0001) and men had a higher prevalence than women, with significant differences in the 40–49 and 60–69 age ranges.

**Table 2:** Age- and gender-specific prevalence of stroke for the study population.

Age (years)	Total		Females		Males		*P value* ^*^
	Number	Stroke cases *(%)*	Number	Stroke cases *(%)*	Number	Stroke cases *(%)*	
40~	2749	18 (0.65)	1615	5 (0.31)	1134	13 (1.15)	**0.0074**
50~	2488	33 (1.33)	1689	20 (1.18)	799	13 (1.63)	0.3672
60~	2016	78 (3.87)	1264	34 (2.69)	752	44 (5.85)	**0.0004**
70~	765	48 (6.27)	369	20 (5.42)	396	28 (7.07)	0.4341
Total	8018	177 (2.21)	4937	79 (1.60)	3081	98 (3.18)	**<0.0001**

^∗^P value is calculated between the two genders.

The factors associated with stroke risk are shown in Table 3. The OR of stroke for a 10-year increase in age was 1.87 (95% CI, 1.58–2.24). The results show that the risk of stroke was two-fold higher among males than among females (OR = 2.03; 95% CI, 1.42–2.90). Participants with a family history of stroke had a four-fold higher risk of stroke than did those without a family history (OR = 4.33; 95% CI, 2.89–6.49). Additionally, hyperlipidemia was significantly associated with stroke risk, and the increased risk was 1.87 (95% CI, 1.31–2.68) among the populations with hyperlipidemia. Moreover, participants with hypertension were at higher stroke risk (OR = 1.47, 95%CI, 1.02–2.12). Finally, physical inactivity was associated with a significantly increased risk of stroke. Compared with those exercising ≥ 3 times per week, participants who reported engaging in physical activity < 3 times per week had a 74% increased risk of stroke (OR = 1.74, 95%CI 1.16–2.59).

**Table 3:** Logistic regression analysis for the association with stroke risk among populations^*^.

Variables	OR 95%CI	*P value*
Age^†^	1.87 (1.58–2.24)	**<0.0001**
Gender		
Female	1.00 (ref)	
Male	2.03 (1.42–2.90)	**0.0001**
Family history of stroke		
No	1.00 (ref)	
Yes	4.33 (2.89–6.49)	**<0.0001**
History of hyperlipidemia		
No	1.00 (ref)	
Yes	1.87 (1.31–2.68)	**0.0007**
History of hypertension		
No	1.00 (ref)	**0.04**
Yes	1.47 (1.02–2.12)	
Physical inactivity		
No	1.00 (ref)	**0.007**
Yes	1.74 (1.16–2.59)	

^†^Odds ratio for each additional decade of age.

^*^Adjustment for education level, marital status, BMI, health insurance status, smoking status, alcohol intake, history of diabetes, heart disease and atrial fibrillation, and other variables in the model.

Abbreviations: OR, odds ratio.

The variables that were significantly and independently associated with ischemic stroke were age, male gender, family history of stroke, history of hyperlipidemia, history of hypertension, and physical inactivity. The factors independently associated with hemorrhagic stroke were being male and having a family history of stroke (Table 4).

**Table 4:** Results of multivariable analyses for stroke types^*^.

Variables	Ischemic stroke (n = 136)		Hemorrhagic stroke (n = 27)	
	*OR 95%CI*	*P value*	*OR 95%CI*	*P value*
Age^†^	1.93 (1.55–2.39)	<0.0001		
Gender				
Female	1.00 (ref)		1.00 (ref)	
Male	2.16 (1.44–3.25)	0.0002	2.52 (1.04–6.12)	0.04
Family history of stroke				
No	1.00 (ref)		1.00 (ref)	
Yes	3.96 (2.49–6.31)	<0.0001	3.31 (1.19–9.21)	0.02
History of hyperlipidemia				
No	1.00 (ref)			
Yes	1.85 (1.23–2.80)	0.003		
History of hypertension				
No	1.00 (ref)	0.04		
Yes	1.53 (1.01–2.33)			
Physical inactivity				
No	1.00 (ref)	0.01		
Yes	1.79 (1.14–2.80)			

^†^Odds ratio for each additional decade of age. *Adjustment for education level, marital status, body mass index, health insurance status, smoking status, alcohol intake, history of diabetes, heart disease and atrial fibrillation, and other variables in the model. Abbreviations: OR, odds ratio.

## Discussion

This study investigated the prevalence of stroke and relevant determinants among middle-aged and older Chinese adults in Shenzhen, China, and the results indicated that the overall prevalence of stroke in adults aged ≥40 years was 2.21%. In previous studies, the prevalence rate of stroke has been estimated at 1.17–9.30%^5–10^ in some areas of China. Notably, these studies were not based on a population-based survey of middle-aged and older Chinese adults. The prevalence of stroke ranges from 0.55% to 6.40% in Saudi Arabia^11^, Singapore^12^, the United States (US)^1^, India^13^, Thailand^14^, Korea^15^, Spain^16^, and Italy^17^. The differences might be at least partly attributable to the participants’ characteristics, including their age, gender, socio-economic status, geographic regions, and sample size.

This study examined the common set of potential risk factors associated with stroke, and some valuable findings were identified. First, stroke was more common among males than among females. Similar findings have been reported in China^10,18^ and other countries, such as German^19^, the United Kingdom^20^, the US^21^, Italy^17^, Spain^16^, New Zealand^22^, and Argentina^23^. More importantly, the multivariable analyses indicated that gender was a significant indicator of stroke; men were more than two times more likely to develop stroke than women. One possible explanation could relate to differences involving genetic factors. Another possible interpretation concerns the protective effects of estrogen on cerebral circulation^24^. Previous studies have shown that lifetime exposure to ovarian estrogens might protect against ischemic stroke^25^. In addition, males have a higher prevalence of hypertension, ischemic heart disease, and smoking, which have been established as risk factors for cerebrovascular disease^26–31^. These results suggest that males should focus more on stroke prevention, and they provide insight for future research on how the biological mechanisms of stroke are affected by gender. Additional studies are warranted to investigate the potential biological mechanism and differences between the sexes.

The results of our study suggest that hyperlipidemia is an independent risk factor for ischemic stroke, similar to many other studies^10,11,32,33^. It is recommended that health administrators and other related departments focus on strengthening the implementation of early intervention strategies. Consequently, the provision of more information on the disadvantages and risk of hyperlipidemia would improve public perceptions of controlling blood cholesterol, thereby reducing the risk of stroke.

Similar to many other studies^10,11,33,34^, our study also found that hypertension was an important indicator of stroke. As such, efforts to improve the treatment of patients with hypertension are necessary to reduce stroke risk. Therefore, health departments and related institutions should conduct regular BP screening and improve treatment adherence through additional community programs to reduce the risk of stroke.

In addition, this study showed that physical inactivity was an important predictor of stroke, which was consistent with previous studies^35,36^. In our study, only 15.97% of participants reported participating in physical activity, and this figure was low. There is a need to understand the behavioral concepts of physical activity, develop appropriate health education materials, and create general awareness about the harmful effects of sedentary behavior. Routine physical activity should be recommended to prevent stroke in community health education and promotion programs.

Interestingly, previous studies identified that obesity was an important risk factor for stroke^37–39^, but in our current study, we found obesity to be a significant risk factor in only univariate analysis (data not shown), not in multivariable analysis. In further analyses, when we stratified BMI into <22.9, 23.0–24.9 and ≥25.0 kg/m^2^ (the proposed cut-offs for normal weight, overweight and obesity for Asian populations), the association between obesity and stroke risk remained non-significant in multivariable analysis (data not shown). This non-significant finding may be attributable to the small number of cases in the present study, which limited the power of the analysis. More studies to replicate our findings are needed.

To the best of our knowledge, this is the first community-based cross-sectional study to investigate the prevalence of stroke and relevant determinants among middle-aged and older adults in China based on the CSPP. Secondly, we found that a much higher likelihood of stroke risk for men than for women.

However, this study had some limitations that must be acknowledged. First, the prevalence could have been underestimated. Some stroke cases may not have been identified through the screening questionnaire or patients may have died before they could be interviewed, resulting in underestimation of the prevalence. Second, this survey was a cross-sectional study, which could restrict the interpretation of the observed associations in terms of causality. Additional community-based prospective studies to confirm the present findings are thus warranted. Third, the generalization of the data to other populations in China, particularly other racial groups, and to other poor regions may be limited. Hence, studies with larger samples and population-level data from both urban and rural areas could provide better estimates of the prevalence and risk factors for stroke in China. Finally, other factors, such as the awareness of stroke warning signs, living status, sleep duration and quality, medications dosage, and dietary and psychosocial factors were not measured in the survey, but these might also be significant risk factors for stroke.

## Conclusion

In conclusion, this study shows that the prevalence of stroke among Chinese adults aged ≥ 40 years is 2.21%. The prevalence, which rises with age, is higher among individuals with a family history of stroke, males, individuals with hyperlipidemia, individuals with hypertension, and physically inactive individuals. These findings provide insight for the healthcare sector on the need to formulate higher-priority strategies for the primary prevention of stroke in China.

## Methods

### Study population

The community-based cross-sectional study was conducted between January 2014 and June 2015 in Bao’an district, Shenzhen, China. Residents were invited to participate if they met the following criteria: (1) participants were adults aged 40 years or older, and (2) participants agreed to complete the CSPP survey. Researchers selected the appropriate participants in the screening process according to these inclusion criteria. Initially, 8,555 adults aged ≥40 years lived in the community of Taoyuan Ju of Bao’an district. All participants were registered at the local government office. Of these adults, 8,043 received the stroke screening, yielding a response rate of 94.13%. Additionally, 35 questionnaires were discarded because information on stroke status was missing. Finally, 8,018 eligible participants were included in the analysis.

This study was conducted in accordance with the principles of the Declaration of Helsinki and approved by the institutional review boards of Tongji Medical College, Huazhong University of Science and Technology, Wuhan, China. All participants read a statement that explained the purpose of the survey and provided written informed consent before participation in the study.

### Data collection

Data were collected by a neurologist and trained medical staff using a standardized questionnaire upon exit from the community health center. The questionnaire used in the present study, was derived from the CSPP. Participants were asked by trained medical staff to provide information regarding their stroke history and the status of risk factors. All data were collected by a neurologist or a trained physician through a structured questionnaire to obtain detailed information, including demographic data, stroke history, diagnosis date, clinical manifestations of claimed acute stroke, lifestyle risk factors, family history of chronic diseases, and status of risk factors. Physical examinations included the assessment of height, weight, and blood pressure (BP) and an electrocardiogram (ECG). Laboratory examinations included the measurements of serum lipids (total cholesterol, low-density lipoprotein cholesterol (LDL-C), high-density lipoprotein cholesterol (HDL-C), triglycerides) and fasting plasma glucose (FPG). The senior investigators checked the collected questionnaires daily as a quality control measure. The data were entered in a double-blind manner to the database by two different researchers using EpiData 3.0 to guarantee accuracy.

### Ascertainment of stroke history and risk factors

Stroke history was evaluated through the combination of self-reporting and the judgment of a neurologist or physician according to the World Health Organization (WHO) MONICA Project definition^40^. Participants with a history of only transient ischemic attack were excluded. The defining criterion of risk factors was guided by the Adult Treatment Panel III and the WHO^41,42^. Smoking was defined as smoking ≥ 1 cigarette per day in the last 3 months. Alcohol drinking was based on self-reports of drinking by ≥ 100 ml spirit alcohol more than three times per week. BMI was calculated as weight in kilograms divided by height in meters squared (kg/m^2^). Predefined categories of BMI (normal weight < 25, overweight 25–30, and obesity ≥ 30 kg/m^2^) were based on the BMI classification for adults defined by the WHO^43^. Hypertension was defined as resting systolic blood pressure (SBP) ≥ 140 mmHg, and/or diastolic blood pressure (DBP) ≥ 90 mmHgor defined by the use of antihypertensive drugs^44,45^. Hyperlipidemia was identified for individuals taking antilipemic drugs or having one or more of the following: TC ≥ 5.2 mmol/L, LDL-C ≥ 3.4 mmol/L, HDL-C ≤ 1.04 mmol/L, or TG ≥ 1.70 mmol/L^46^. In our study, diabetes mellitus was defined as FPG ≥ 7.0 mmol/L or defined by the use of diabetes medication^47^. In this survey AF was identified for those with a history of persistent AF or defined based on past electrocardiogram (ECG) or ECG examination results. Physical activity was defined (based on self-reports) as ≥ 3 times per week for at least 30 minutes at a time.

### Statistical analysis

Descriptive analyses included means for the continuous variables and percentages for the categorical data. Student’s *t*-test was used to compare variable means between participants with and without stroke, and the *χ*^2^ test was used for the categorical variables. Multivariable stepwise logistic regression analysis was used to examine the associations between stroke and independent variables. We used the stepwise selection method to select variables that were associated with stroke risk (level for selection and elimination: *P* = 0.05 and *P* = 0.10 respectively). In the multivariable model, the independent variables included age in 10-year intervals, gender (female and male), marital status (single, widowed, divorced, married), education level (primary, junior, senior, college), BMI (<25, 25–29, 30+ kg/m^2^), health insurance (yes, no), family history of stroke (yes, no), history of diabetes (yes, no), history of hypertension (yes, no), history of hyperlipidemia (yes, no), history of heart disease (yes, no), history of AF (yes, no), smoking status (never, former, current smoker), alcohol drinking (never, occasionally, frequently), and physical activity (yes, no). We also restricted our analysis to identify factors associated with ischemic and hemorrhagic stroke separately. All statistical procedures were performed using the Statistical Analysis System (SAS) 9.2 for Windows (SAS Institute Inc., Cary, NC, USA), and the statistical tests were two-tailed with a significance level of 0.05.
