# Author Correction: Prevalence and risk factors associated with stroke in middle-aged and older Chinese: A community-based cross-sectional study

**DOI:** 10.1038/s41598-018-22785-w

**Published:** 2018-03-09

**Authors:** Yong Gan, Jiang Wu, Shengchao Zhang, Liqing Li, Xiaoxv Yin, Yanhong Gong, Chulani Herath, Naomie Mkandawire, Yanfeng Zhou, Xingyue Song, Xiaozhou Zeng, Wenzhen Li, Qiaoyan Liu, Chang Shu, Zhihong Wang, Zuxun Lu

**Affiliations:** 1 Department of Social Medicine and Health Management, School of Public Health, Tongji Medical College, Huazhong University of Science and Technology, Wuhan, Hubei China; 2 Bao’an Central Hospital of Shenzhen, Shenzhen, Guangdong China; 3 Department of Management, School of Economics and Management, Jiangxi Science and Technology Normal University, Nanchang, Jiangxi China; 4 Department of Epidemiology and Biostatistics, School of Public Health, Tongji Medical College, Huazhong University of Science and Technology, Wuhan, Hubei China; 5 Department of Neurosurgery, Shenzhen Second People’s Hospital, Shenzhen University, Shenzhen, Guangdong China

Correction to: *Scientific Reports* 10.1038/s41598-017-09849-z, published online 25 August 2017

This Article contains an error in the Acknowledgements section

“We thank all staff members involved in this study for their painstaking efforts in conducting the data collection. This study was supported by the Fundamental Research Funds for the Central Universities, Huazhong University of Science and Technology, China (2016YXMS215).”

should read:

“We thank all staff members involved in this study for their painstaking efforts in conducting the data collection. This study was supported by Natural Science Foundation of China (NSFC, 71373090, “Study on the gatekeeper policy of community health service).”

